# Multiclass Classification of Visual Electroencephalogram Based on Channel Selection, Minimum Norm Estimation Algorithm, and Deep Network Architectures

**DOI:** 10.3390/s24123968

**Published:** 2024-06-19

**Authors:** Tat’y Mwata-Velu, Erik Zamora, Juan Irving Vasquez-Gomez, Jose Ruiz-Pinales, Humberto Sossa

**Affiliations:** 1 Robotics and Mechatronics Lab, Centro de Investigación en Computación, Instituto Politécnico Nacional (CIC–IPN), Avenida Juan de Dios Bátiz esquina Miguel Othón de Mendizábal Colonia Nueva Industrial, Vallejo CP, Gustavo A. Madero, Mexico City 07738, Mexico; tmwata@cic.ipn.mx (T.M.-V.); hsossa@cic.ipn.mx (H.S.); 2 Section Électricité, Institut Supérieur Pédagogique Technique de Kinshasa (I.S.P.T.-KIN), Av. de la Science 5, Gombe, Kinshasa 03287, Democratic Republic of the Congo; 3 Telematics and Digital Signal Processing Research Groups (CAs), Department of Electronics Engineering, Universidad de Guanajuato, Salamanca 36885, Mexicopinales@ugto.mx (J.R.-P.); 4 Centro de Innovación y Desarrollo Tecnológico en Cómputo, Instituto Politécnico Nacional, Avenida Juan de Dios Bátiz esquina Miguel Othón de Mendizábal Colonia Nueva Industrial, Gustavo A. Madero, Mexico City 07738, Mexico; jvasquezg@ipn.mx

**Keywords:** brain–computer interfaces (BCIs), visual EEG classification, mutual information (MutIn), minimum-norm estimate (MNE), EEGNet, convolutional neural network (CNN), long short-term memory (LSTM)

## Abstract

This work addresses the challenge of classifying multiclass visual EEG signals into 40 classes for brain–computer interface applications using deep learning architectures. The visual multiclass classification approach offers BCI applications a significant advantage since it allows the supervision of more than one BCI interaction, considering that each class label supervises a BCI task. However, because of the nonlinearity and nonstationarity of EEG signals, using multiclass classification based on EEG features remains a significant challenge for BCI systems. In the present work, mutual information-based discriminant channel selection and minimum-norm estimate algorithms were implemented to select discriminant channels and enhance the EEG data. Hence, deep EEGNet and convolutional recurrent neural networks were separately implemented to classify the EEG data for image visualization into 40 labels. Using the k-fold cross-validation approach, average classification accuracies of 94.8% and 89.8% were obtained by implementing the aforementioned network architectures. The satisfactory results obtained with this method offer a new implementation opportunity for multitask embedded BCI applications utilizing a reduced number of both channels (<50%) and network parameters (<110 K).

## 1. Introduction

Brain–computer interfaces (BCIs) based on electroencephalographic (EEG) signals are gaining considerable attention in scientific research and application development [1] because of technological advances and multidisciplinary studies related to brain signals [2,3]. Many categories of EEG signals can be processed, and countless BCI systems have been developed for ordinary use and clinical applications, for example, in the fields of brain-controlled vehicles [4], drones [5], assistive devices [6], intelligent systems [7], neurorehabilitation [8], telemedicine [9], assistive robots [10], and wheelchairs [11], to name but a few. Based on a user’s mental stimulation, event-related potentials (ERPs) are generated externally by cognitive load or auditory, sensory, or visual stimuli. In addition, ERPs are involved in internal processes such as stress, directed thought, and memory and concentration [12]. In particular, the visually evoked potentials (VEPs) considered in this work are sensory potentials induced by visual stimuli [13]. In several of the aforementioned BCI applications, EEG signals have their features tagged as class labels, which is an advantage when controlling several tasks, according to the BCI system’s functioning logic. A complex BCI system needs more class labels than a classical one. In this sense, to control an assistive robotic arm using motor EEG signal imagery, Onose et al. [14] used four mental tasks that were randomly distributed into sequences of 35 trials each, while Zhu et al. [15] controlled a robotic arm with six degrees of freedom using 15 target classes. Statistically, a ranking metric uses the probability that an input data stream belongs to one of the implemented model’s output labels. The more labels there are to classify, the less likely a data sequence will be correctly classified. Therefore, in order to take advantage of complex BCI systems, classifying EEG signals into multiple classes is necessary.

However, as is mentioned by Del Moral et al. [16], doing this significantly increases the number of classes, which makes the computational task of properly classifying a new feature vector into one of the classes a challenge.

In addition, EEG signals naturally carry their own inherent processing challenges, as they are produced by nonstationary mental states [17]. The nonstationarity and nonlinearity characteristics of EEG signals mean that the processing algorithms for BCI systems must be rigorous and designed with an outstanding level of precision [18].

To address the challenge of accurately classifying EEG signals into multiclass labels for BCI systems, various algorithms based on robust model architectures have recently been proposed as a result of newly available public databases. Mahmood et al. [19] proposed a multiclass classification algorithm based on the Common Spatial Pattern (CSP) and support vector machine (SVM) for BCI applications. They achieved an average accuracy of 85.5% using four frequency bands to classify motor EEG signal imagery into four classes. Recently, for five-class classification, motor EEG signal imagery was processed as three-channel images using Deep Convolutional Neural Networks (DCNNs) and long short-term memory (LSTM) networks [20]. The model achieved an average accuracy of 70.64% using the Physionet dataset for EEG motor imagery tasks. Another innovative approach to improve BCI performance in multiclass classification consists of combining two different BCI modalities. On this basis, Kwon et al. [21] implemented a compact hybrid BCI based on EEG and fNIRS using the channel selection and source–detector (SD) pair approaches. A high classification accuracy of 77.6% was obtained in classifying three mental states. To address the challenge of multiclass classification in BCI systems, Spampinato et al. [22] released an EEG signal database captured from six subjects visualizing image sequences. Initially, using recurrent neural networks (RNNs) and convolutional neural networks (CNNs) to learn and classify visual stimuli-evoked EEG signals from 40 ImageNet object classes, an average accuracy of 82.9% was achieved. Next, focusing on decoding visual information from brain signals with the same database and a multimodal approach based on joint learning, Palazzo et al. reported accuracies of 60.4, 50.3, 54.7, 46.2, and 60.4% when employing the Inception-V3, ResNet-101, DenseNet-161, AlexNet, and EEG-ChannelNet networks, respectively.

## 2. Related Work

Currently, research on modeling complex cognitive events from EEG signals is attracting great interest due to the flourishing deployment of BCI systems. As assistive devices, BCI systems based on EEG signals are designed to meet the user’s requirements in terms of convenience and adaptability. Generally, EEG signal processing for BCI applications considers the contribution of channel selection according to the defined paradigm, e.g., for noise reduction, artifact removal [23], feature extraction, or classification. It should be pointed out that some recent algorithms can merge two or more functions [24]. Channel selection algorithms aim to identify the optimized subset of EEG electrodes; however, the channels that are not activated by the mental task can introduce noise and, thus, negatively impact the classification results [25]. Additionally, the computational complexity can be reduced by selecting only those channels related to the mental task, resulting in faster real-time processing. In this sense, Alotaiby et al. [26] proposed an interesting review of channel selection algorithms for EEG data processing, emphasizing filtering, wrapper, embedded, hybrid, and human-based techniques. Among the filtering approaches, variance-based, difference in variance-based, and entropy-based selection have been commonly implemented in the recent literature [27,28]. For their part, regression-based approaches, filtering algorithms, and blind source separation-based techniques have been predominantly used to improve EEG by reducing noise [29]. The MNE-Python library, which was recently released by Gramfort et al. [30], provides suitable tools to enhance EEG/EMG signals, among other functionalities. Lastly, taking advantage of recent advances in machine and deep learning, EEG feature extraction and classification provide BCI systems with distinctive and useful attributes. For instance, Yedukondalu and Sharma [31] implemented K-nearest neighbors (KNN) and support vector machine (SVM) classifiers to identify cognitive load during mental arithmetic tasks, achieving an accuracy of 96.88%. In parallel, EEG signals from six auditory stimuli were classified for BCI applications utilizing classifiers based on random forest, multilayer perceptron, and decision tree architectures [32], wherein average accuracies of 91.56%, 89.92%, and 86.78% were reported, respectively. In addition, Kalafatovich et al. implemented a two-stream convolutional neural network to classify single-trial EEG signals evoked by visual stimuli into two and six semantic categories [33]. They achieved accuracies of 54.28 ± 7.89% for the six-class case and 84.40 ± 8.03% for the two-class case. Recently, EEG signals induced by visual stimuli evoked by 40 image classes of the ImageNet dataset were classified using an RNN and CNN [22]. A maximum accuracy of 82.9% was achieved by classifying EEG signals corresponding to image sequences. That work addressed the recent challenge of EEG multiclass classification by offering a reliable alternative for multitask BCI-based applications. Indeed, for BCI-based robotic applications, for example, a BCI system based on multitasks allows the robot’s degree of freedom to be covered, whereby each mental task controls the robot’s specific movement. In this manner, taking advantage of the PL dataset availability, Zheng and Chen proposed attention-based Bi-LSTM models for classifying EEG signals evoked by image visualization into 40 classes [34]. Among other results, they reported classification accuracies of 96.27% and 99.50% using 29 prefrontal and occipital channels, and all 128 dataset channels, respectively. However, their models used more than 300,000 network parameters to achieve the aforementioned results. In addition, they partitioned the 500 ms EEG sequence into visualization time segments of 40–200, 40–360, and 360–480 ms to evaluate the classification accuracy improvement. The PL dataset was also used in [35], where a combination of ensemble and deep learning models allowed category-dependent representations of EEG signals to be extracted. The proposed LSTMS_B model achieved an average accuracy of 97.13%, classifying visual EEG segments into 40 classes. The authors did not report the number of training parameters required by their model. Another recent approach using the PL dataset was proposed by Kumari et al. [36]. EEG signals evoked from visual stimuli were processed as spectrogram images using a Capsule Network (EEGCapsNet) based on Short-Term Fourier Transform (STFT). An average accuracy of 81.59% was reported, classifying EEG representations into 40 classes. Similarly, the number of network parameters was not precisely reported. Finally, a functional connectivity-based geometric deep network (FC-GDN) was proposed by Nastaran et al. to classify EEG recordings evoked by images into 40 classes [37]. They obtained an average accuracy of 98.4% by configuring their model with at least 600,000 training parameters. Table 1 presents studies in the recent literature in which the PL dataset was used.

To address the challenge of visual EEG multiclass classification, in the present work, we explore the use of a reduced number of channels and two deep learning networks. Concretely, a mutual information method based on cross-entropy is developed, allowing the grouping of discriminant channels. Therein, once the set of 54 discriminant channels is constituted, the preprocessing step is carried out by the minimum-norm estimates algorithm (MNE-Python) [30,38]. Regarding the classification step, the proposed method uses two classifiers with optimized numbers of network parameters. The first approach implements the EEGNet network, which has demonstrated excellent performance in the literature because of its temporal and spatial filter banks [39,40]. The second approach uses a combination of a CNN and LSTM network to extract and classify EEG features into 40 targeted classes. Therefore, the PL dataset published in [22] and the k-fold cross-validation technique were used to assess the proposed method. The paper’s contributions are summarized as follows:

1. A visual multiclass classification approach based on both a reduced number of channels and network parameters is proposed for BCI applications.
2. Comparative results for the EEGNet and CNN-LSTM classifiers using the PL dataset EEG data are presented.
3. A channel selection approach based on mutual information is implemented to accurately discriminate contributing channels.

The results achieved in this paper offer new possibilities for multitask embedded BCI applications based on EEG signals. After the Introduction and Related Work sections, the remainder of the article is organized as follows. The method developed in this work is outlined in Section 3, including a high-level general diagram, signal enhancement, and the classifier models. Section 4 presents the dataset employed in this work. Finally, the results obtained are reported and discussed in Section 5, and Section 6 provides the paper’s conclusion and explores potential future work.

## 3. Methods

The proposed method focuses on accurate EEG signal processing, emphasizing the mutual information (MutIn) approach to select discriminant channels and the MNE algorithm to enhance signals. Next, EEGNet and CNN-LSTM classifiers are used to estimate the output class label probability. In other words, with this method, we aimed to classify EEG signals from a reduced number of channels, while providing a reliable classification accuracy. Because the capture system uses 128 electrodes, organized according to the 10–20 system [41], and considering brain cortex-specific functions [42], selecting discriminant channels allows a channel subset that optimizes the classifier performance to be established. Once the discriminant channel subset is constituted, EEG data are preprocessed using the MNE-Python package [30]. This is essentially for the time–frequency analysis. This signals analysis aims to efficiently make the data more suitable for classifiers by inspecting artifacts in both time windows and frequency patterns. In the final step, EEGNet and CNN-LSTM networks configured with an optimal number of parameters are separately used to extract and classify feature data.

### 3.1. Overall Flowchart

**Figure 1:**
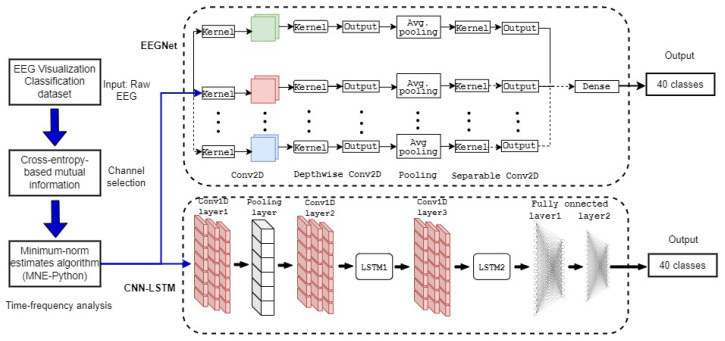
High-level general diagram of the proposed method. The EEG signal visualizations from 128 channels were obtained from the public dataset published in [22]. Next, 64 channels were selected by evaluating the channels’ MutIn. Finally, the MNE algorithm was applied to enhance the EEG data, which were classified into 40 labels separately by the EEGNet and CNN-LSTM architectures.

Figure 1 presents the high-level general diagram of the proposed method. Four gradual steps were utilized in the method development. The first consisted of downloading and preparing data from the referenced dataset. In the second stage, a channel selection approach based on MutIn was developed to build a subset of 64 discriminating channels [26,43]. Next, the minimum-norm estimates (MNE) algorithm implemented in Python language was used to preprocess data from selected channels. Finally, the EEGNet and CNN-LSTM models classified EEG segments into 40 classes, separately providing outputs.

### 3.2. Mutual Information-Based Channel Selection

The selection of discriminant channels related to the defined mental task acted to gather channels delivering similar information based on entropy values. In the recent literature, similar methods have been proposed to select contributing channels, particularly in [43,44,45]. In this sense, let M=[1,2,i,⋯,j,⋯,128] be the finite channel set provided by the dataset, and $A_{i}$ and $B_{j}$ be two probability distributions of channels *i* and j∈M. The Kullback–Leibler Divergence (KLD) assesses how far a signal joint distribution of *i* and *j* channels is from the probability distribution of their products. It is calculated as follows:

(1) $$\mathrm{KLD}\left(A_{i}\;∥\;B_{j}\right)=\sum_{a\;\in \;M}A_{i}\left(a\right)\log\frac{A_{i}\left(a\right)}{B_{j}\left(a\right)},$$

where $A_{i}\left(a\right)$ is the occurrence probability of the $a^{th}$ information. Thus, the MutIn between channel pairs is found by evaluating the KLD as follows:

(2) $$\begin{array}{cc} I(M_{i};M_{j}) & =KLD(P\left(M_{i},M_{j}\right)\;∥\;P\left(M_{i}\right)P\left(M_{j}\right)), \end{array}$$

where $P(M_{i})$ and $P(M_{j})$ are signal distributions of the $M_{i}$ and $M_{j}$ channels, respectively, and $P(M_{i},M_{j})$ is their joint distribution. Generally, calculating the MutIn using Equation (2) has two outcomes:

- $M_{i}$ and $M_{j}$ are independent; therefore,
  (3) $$\begin{array}{cc} I(M_{i};M_{j}) & =KLD(P\left(M_{i},M_{j}\right)\;∥\;P\left(M_{i}\right)P\left(M_{j}\right))=0; \end{array}$$
- In the other cases, the $M_{i}$ and $M_{j}$ channels share the totality of their respective information. Thus,
  (4) $$\begin{array}{cc} I(M_{i};M_{j}) & =KLD\left(P\left(M_{i},M_{j}\right)\;∥\;P\left(M_{i}\right)P\left(M_{j}\right)\right)=H\left(M_{i}\right), \end{array}$$

where $H(M_{i})$ represents the self-entropy of channel $M_{i}$. In this work, creating a subset of 64 discriminant channels constitutes a novel method as is discussed in Section 5.4. Therefore, when computing Equation (2) according to Algorithm 1, subsets of discriminant channels are constituted by finding maxima cross-entropy values for each considered channel combination.
**Algorithm 1:** MutIn algorithm-based discriminant channel selection.
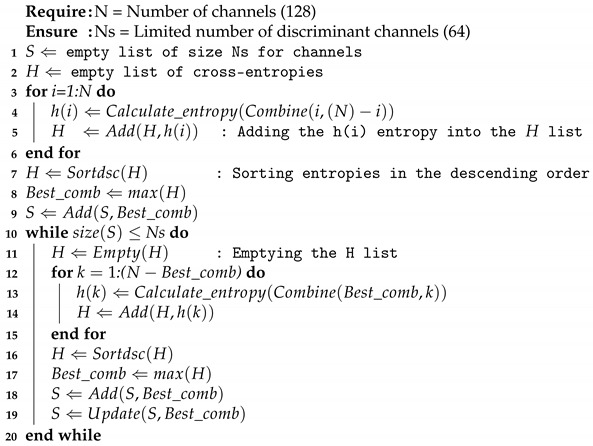


The channel selection step typically involves all dataset signals. This empirical approach ensures the constituted discriminant channel subset is more representative of all subject signals. In addition, this strategy helps in the cross-subject results comparison. From Algorithm 1, lines 3 to 6 calculate the entropies by combining the 128 channels in a two-by-two structure. Next, on lines 10 to 20, a channel combination with a high entropy value is used to make n-channel combinations by adding one discriminant channel in each iteration until the last 64-channel combination is obtained.

### 3.3. Enhancing Signals Using the Minimum-Norm Estimates Algorithm

In the literature related to EEG signal processing, the MNE algorithm and its variants are used more for brain source localization [46,47], estimating the functional connectivity between different brain cortices [30], and EEG inverse problems [48] than for signal preprocessing [49,50]. Typically, the MNE-Python preprocessing pipeline allows for an EEG quality assessment provided by selected channels. As a result, EEG segments are extracted using band-stop, band-pass, low-pass, high-pass, and notch filtering [30].

In the present work, MNE-Python’s semi-automatic functions were implemented to exclude contaminated EEG data and reduce artifact attenuation. Algorithm 2 summarizes the relevant steps in EEG data preprocessing using the MNE.
**Algorithm 2:** The MNE steps implemented to enhance the EEG data.**1** Obtain EEG data from selected channels.**2** Render poor channels providing extremely noisy data usable, based on good signals delivered by other channels.**3** Discard erroneous data gaps and spans.**4** Calculate the variance of the data.**5** Remove the mean and scale of the unit variance to standardize features.**6** Create epoch of data.**7** Average epochs to obtain evoked responses.

The class *mne.decoding.Scaler*, which includes steps 1 to 5 of Algorithm 2, was specifically utilized. This class estimates the mean (μ) and variance (σ) for each channel by utilizing data from all epochs and time points. That is, the μ and σ of a given $n^{th}$ training sample are estimated as follows:

(5) $$\mu_{n}\approx \frac{1}{N}\left(\sum_{i=1}^{N}x_{i}\right),\;\;\mathrm{and}$$

(6) $$\sigma_{n}^{2}\approx \frac{1}{N-1}\sum_{i=1}^{N}{\left(x_{i}-\mu_{n}\right)}^{2},$$

where *N* is the number of epochs, and *i* denotes each epoch’s time points. Therefore, each feature is independently centered and scaled by computing the corresponding statistics on the training set’s samples. By setting $\mathrm{scalings}{=}^{\prime }{\mathrm{mean}}^{\prime }$, all other parameters related to the previously mentioned function are used with their default values as explained in the implementation documentation [30]. Next, data are fit by standardizing them across channels. Then, they are transformed, coupled to their labels, to produce a new version of the epoch data as the output. Hence, mu and σ are utilized in the data using the MNE *fit_transform()* function. Finally, the obtained epoch is averaged across channels to obtain evoked responses, covering steps 6 to 7 in Algorithm 2. According to the data provided by the PL dataset presented in Section 4, a total of 440 epochs were created per trial and subject, corresponding to the visualization of 40 visual stimuli. Therefore, it was assumed that 11 epochs were generated per class for the time lock of 440 ms. Next, for the time-window analysis, nine other epoch groups were created to evaluate the classifiers’ performance, namely, 20–240, 20–350, 20–440, 40–200, 40–360, 130–350, 130–440, 240–440, and 360–440 ms.

### 3.4. The Implemented Classifiers

Two deep learning architecture models, namely, the EEGNet and a hybrid CNN-LSTM networks [39,51,52], were considered. They were chosen because of their respective performances in processing EEG signals and the outstanding number of referenced studies.

#### 3.4.1. EEGNet Network

**Figure 2:**
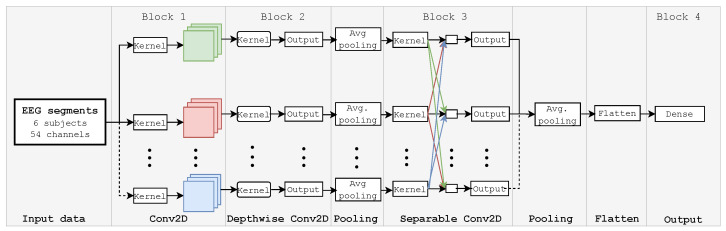
The EEGNet architecture. Conv2D extracts temporal features in the first block. Feature maps are enhanced in Block 2 using spatial filters, which are combined in Separable Conv2D. Finally, Block 4 estimates the output probability for a processed feature map.

Built in Keras and Tensorflow [53,54], EEGNet is a compact convolutional neural network proposed by Waytowich et al. [39] for EEG signal processing and classification. As illustrated in Figure 2, the EEGNet architecture combines three convolutional layers, i.e., a temporal, depthwise, and separable convolution layer.

Epochs from the MNE block are convolved using the Conv2D (Block 1) where frequency filters are applied. Thereafter, each feature map in the Depthwise Conv2D layer (Block 2) is processed by spatial filters to determine its intrinsic properties. Preceded by a pooling layer, depthwise and pointwise convolutions are executed independently in the Separable Conv2D layer (Block 3) before being combined. Finally, subsequent to the pooling and flatten layers, the classification layer (Block 4) evaluates the probability that an output EEG segment belongs to one of the forty input labels. To do this, the Exponential Linear Unit (ELU) function is used to activate the depthwise and separable Conv2D layers as follows:

(7) $$f\left({\vec{x}}_{i}\right)=\left\{\begin{array}{cc} x_{i} & \mathrm{for}\;x\geq 0,\\ \alpha (e^{x_{i}}-1) & \mathrm{otherwise}, \end{array}\right.$$

and, in addition, the dense layer is activated by the Softmax function, which is given by

(8) $$\sigma {\left(\vec{x}\right)}_{i}=\frac{e^{x_{i}}}{\sum_{j=1}^{40}e^{x_{j}}},\;\forall \;\vec{x}={\left[x_{1},x_{2},\ldots ,x_{40}\right]}^{⊺},$$

where $x_{i}$ and $x_{j}$ represent the input and output sequences of time points, respectively. The hyper-parameter α controlling the saturation point for negative inputs is set to 1. Specifically, the Conv2D layer featured eight temporal filters (F1) with 320 network parameters, the Depthwise Conv2D layer included 10 spatial filters (D) with 4320 parameters, and the Separable Conv2D layer possessed 7680 pointwise filters (F2) utilizing 456 parameters. These filters’ values were set considering the data structure (the sampling frequency, the length of samples per task and subject, etc.) and the outcomes of the preliminary training tests, which aimed to optimize the classifier. Table 2 summarizes the layers’ main parameters for the proposed EEGNet model.

In addition to the filter settings, the model was built to receive 440 time points delivered by the 54 selected channels as inputs. Section 5.4 explains why 54 channel were selected rather than the 64 that were intended. The kernel length was set to 40 to match the number of output classes. In addition, the dropout was set to 0.2. Next, the model was compiled using the categorical cross-entropy loss function, the Nadam optimizer, and the accuracy metric, which are defined in the Results section.

#### 3.4.2. The Proposed CNN-LSTM Model

**Figure 3:**
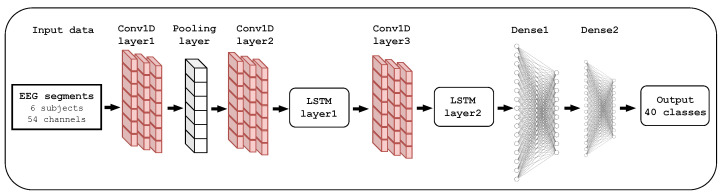
The implemented CNN-LSTM architecture. Two 1D-CNN layers separated by a Max-Pooling layer represent the input block of the model. Next, a cascade of LSTM-1DCNN-LSTM performs feature learning and extraction. Finally, the label output probability is computed by the Softmax layer, which is coupled to the fully connected layer.

The CNN-LSTM model has demonstrated its efficiency in processing EEG signals for application-based BCI systems [55,56]. This architecture finds its greatest use in extracting spatial features at the CNN block level. In addition, temporal dependencies are identified at the LSTM block level as a result of its powerful learning and memory capabilities. Concretely, EEG time points from preprocessing are memorized and forgotten, allowing the model to learn more comprehensive features. Because of memory units in the LSTM block, the CNN-LSTM model can remember the data’s prior state, ensuring identification based on the current state change pattern. At the end of this hybrid process, a fully connected layer guarantees the labeled output by considering an input data sequence. Figure 3 presents the proposed CNN-LSTM architecture, which comprises three CNN layers, two LSTM units, and fully connected and Softmax layers.

Therefore, the Conv1D_layer1 is configured with 128 convolutions with a 3×3 kernel size, while the Conv1D_layer2 and Conv1D_layer3 layers contain 64 filters with a 3×3 size. The *He* initialization algorithm [57] was used to initialize weights based on a uniform distribution, and the dropout parameter was set to 0.2 for the mentioned layers. All convolutional layers were activated by the leaky Rectified Linear Unit (ReLU) function, which is given by

(9) $$f\left(\alpha ,{\vec{x}}_{i}\right)=\left\{\begin{array}{cc} \alpha x_{i} & \mathrm{if}\;x<0,\\ x_{i} & \mathrm{otherwise},\;\forall \;\vec{x}={\left[x_{1},x_{2},\ldots ,x_{40}\right]}^{⊺} \end{array}\right.$$

where α represent a small positive constant, which was set to 0.005 to compensate for negative net inputs with a small nonzero gradient. The LSTM layers were configured with 64 and 32 memory units to process the time-point sequences from the convolutional layers. Finally, the fully connected layer contained 54 neurons, and the Softmax layer used 40 neurons to predict the class probability of the output sequence. As was the case for the EEGNet model, the categorical cross-entropy loss function, the Nadam optimizer, and the accuracy metric were implemented to compile the model. A parameters summary of the proposed CNN-LSTM model is provided in Table 3.

### 3.5. Experimental Settings

The code implementation of both architectures was developed in Python 3.6 using Keras and Tensorflow. A NVIDIA GTX 2080 Ti GPU-equipped Ubuntu 22.04 desktop computer was used to run the entire project. In addition, to accelerate the learning convergence of the models, the Cyclical Learning Rate (CLR) algorithm [58] was implemented, which also helped to avoid local minima in the learning process. The lower and upper bounds of the learning rates were adjusted to $10^{-3}$ and $10^{-7}$, respectively, by adopting a triangular window, and the step size was set to eight times the epoch’s total iterations. The EEGNet and CNN-LSTM models were trained for 1000 epochs for comparison purposes, with a batch size of 440. Finally, the k-fold cross-validation approach presented in Section 5 was used to evaluate the results obtained in this study.

## 4. The Perceive Lab Dataset

**Figure 4:**
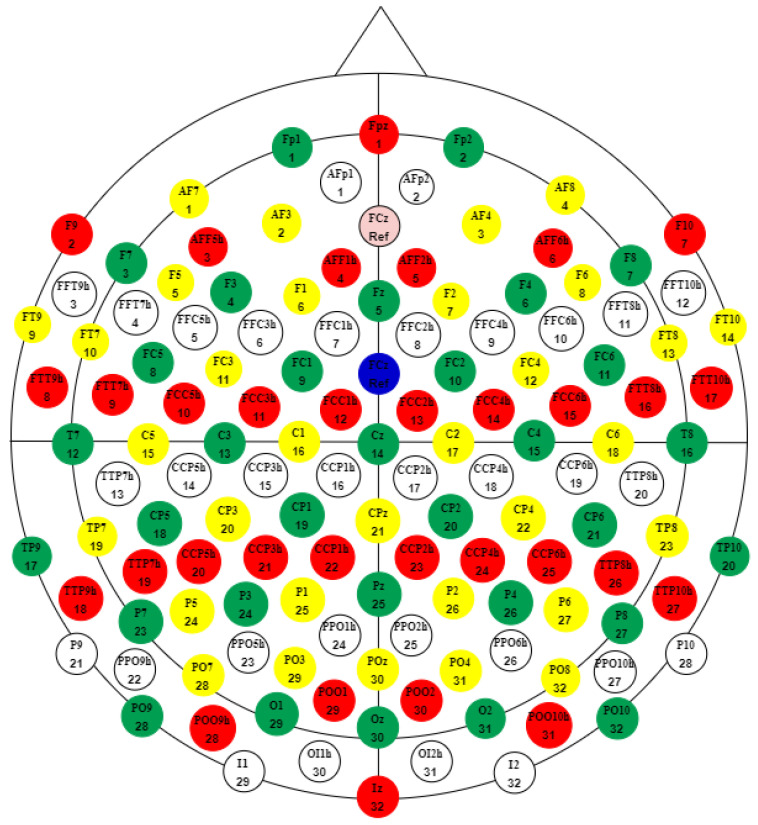
The EEG actiCAP 128-channel standard layout used for the experiment protocol, modified from [60]. A total of 128 electrodes are illustrated in four colors (green, yellow, red, and white). There are 32 active electrodes for each color group. Capital letters in electrode taxonomy typically indicate the spatial location over the brain cortex: T for temporal, Fp for frontal, C for central, P for parietal, and O for the occipital cortex. A classic combination of two letters means that the electrode is placed over the intermediate area between two brain cortices.

The dataset used in this work was provided by Spampinato et al. [22]. A total of 2000 classic images from the ImageNet dataset [59], representing 40 labels of 50 pictures each, served as the visual stimuli for the six test subjects. The ImageNet dataset provides 40 image labels containing cats, sorrels, elephants, fish, dogs, airliners, brooms, pandas, canoes, phones, mugs, convertibles, computers, fungi, locomotives, espresso, chairs, butterflies, golf, piano, iron, daisy, jacks, mailbags, capuchin, missiles, mittens, bikes, tents, pajama, parachutes, pools, radios, cameras, guitar, guns, shoes, bananas, pizzas, and watches. The experiment protocol consisted of instantly visualizing in a continuous sequence of blocks containing images of each label. The display duration for each image block was 0.5 s. This was followed by 10 s of recording EEG signals corresponding to the previously visualized block, before considering the same cycle for the adjacent image block. Each image block segment contained EEG signals from 128 channels, recorded with Brainvision DAQs equipment at a sampling frequency of 1 kHz. The data resolution was set to 16 bits. As depicted in Figure 4, the electrode map of the system is based on the 10–20 placement system. Therefore, Equation (10) presents the number of EEG signal samples corresponding to each channel:

(10) $$N_{samples}=\left(0.5\;s\right)\times 1\;\mathrm{kHz}=500$$

All channel cues were intentionally inscribed into the 440 samples per task to avoid undesirable signals related to potential interference between the previous and current image blocks. This was also performed for the purpose of pattern length uniformization. Three band-pass filters were applied to the signals constituting the dataset—a second-order Butterworth filter from 5 to 95 Hz, another from 14 to 70 Hz, and the final one from 55 to 95 Hz, all including a notch filter at 50 Hz. A total of 11,964 EEG fragments constitute the current dataset, equaling approximately 2000 segments per subject. The others were excluded due to mediocre recording quality. Table 4 summarizes the number of signal segments per subject contained in the dataset.

Finally, Table 5 presents the main parameters of the PL dataset published by Spampinato et al. [22].

## 5. Results

The results presented in this section essentially derive from the data presented in Section 4 via the *k*-folds cross-validation method, where *k* was set to 10, i.e., the whole dataset was divided into 10 partitions. Nine partitions were employed iteratively for training, while one partition was used to validate the models’ performance. Specifically, for each of the 10 iterations, 10,767 samples were used for training and 1197 samples for validation. This validation technique enabled the models’ efficiency to be assessed for specific data streams or several unpredictable inputs. Additionally, the developed classification approach used the accuracy metric defined by

(11) $$Accuracy=\left(\frac{TP+TN}{TP+TN+FP+FN}\right)\times 100,$$

where TP,TN,FP, and FN represent the true positive, true negative, false positive, and false negative, respectively. In addition, TP corresponds to each *x* feature correctly assigned to label X, while TN represents each *x* feature for labels other than X that were not classified to the X label. Different from FN, FP is related to all features misclassified to the X label. Finally, the effectiveness of the suggested approach was assessed using the confusion matrix metric.

### 5.1. Results Related to Channel Selection

The first step of the developed method consisted in selecting discriminant channels from the 128 channels provided by the dataset. In this study, we explored minimizing the number of channels while preserving good classification accuracy. This was because all current deep learning-based studies use all 128 available channels and only focus on improving the classification accuracy. For embedded BCI systems that have severely constrained computing resources and a low power consumption, this accuracy–data size trade-off is essential [61,62]. Therefore, we proposed using less than half of the available channels by developing Algorithm 1 to select the most discriminating channels. Table 6 presents the results achieved in channel selection.

The developed approach allowed for the selection of eight channels in the brain’s parietal cortex and seven in the occipital and parietal–occipital areas. However, only two channels were found to be discriminating in the frontal–central cortex, compared to three in the frontal–central–central area. In summary, EEG signals from the 54 selected channels were considered for the preprocessing step using the MNE method.

### 5.2. Results of Preprocessing Using MNE

**Figure 5:**
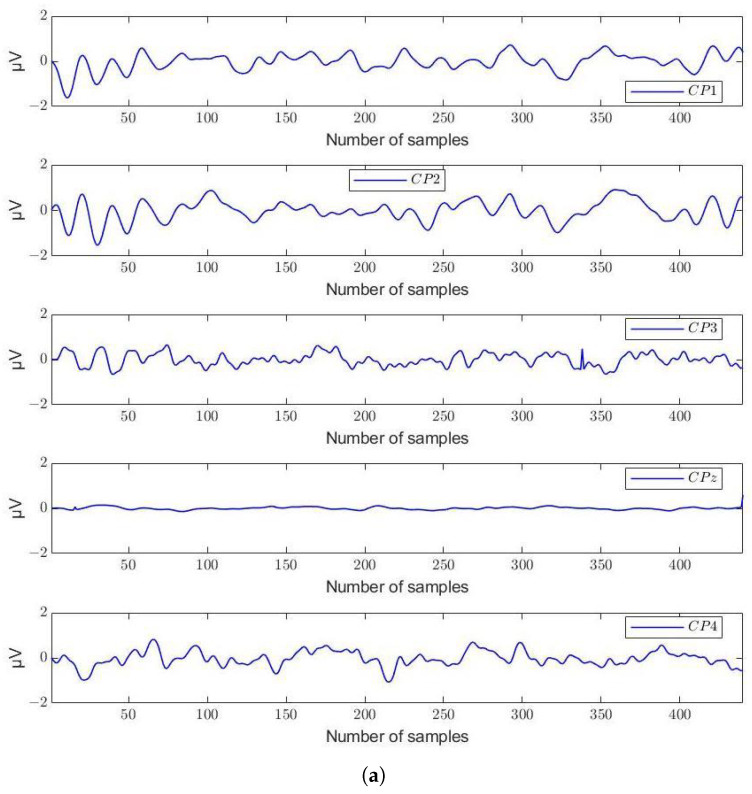

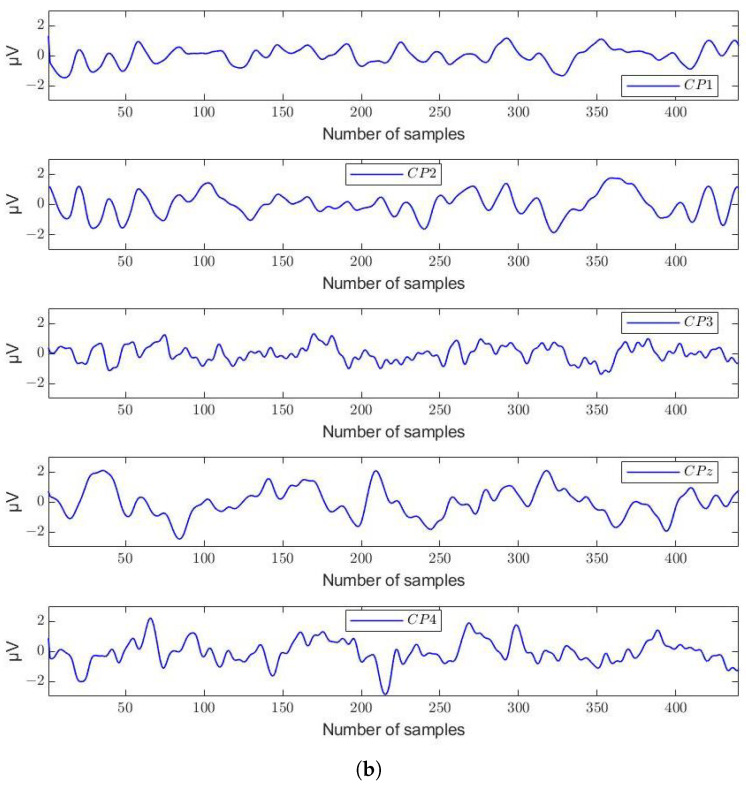
The EEG segments of Subject 4 before and after the application of the MNE algorithm for the data from channels CP1,CP2,CP3,CPz, and CP4. The maximum length of segments is 440. (**a**) Before applying the MNE algorithm. (**b**) After applying the MNE algorithm.

Each EEG segment of the 54 selected channels was enhanced using the MNE algorithm. Therefore, by computing Equations (5) and (6), the length of the epoch data was maintained to 440 at output. As an illustration, Figure 5 presents the EEG segments from Subject 4 for a selected group of discriminant channels, before and after implementing the MNE algorithm.

Thus, the new version of the epoch data illustrated in Figure 5b conserved the class labels of the initial data, but artifacts were removed using the MNE mean scaling procedure. In summary, the data matrix of 11,964 time-point segments from 54 selected channels, containing a maximum of 440 samples, was used as input to the classifiers.

### 5.3. Results Related to EEG Segment Classification

EEG time-point segments were classified with the two approaches. The first approach used a total length of 440 samples. Table 7 presents the results achieved by classifying data with the aforementioned models.

The classifiers’ accuracy is reported for every $k^{th}$-fold iteration, alongside the number of segments utilized for testing and training. Average accuracies of 93.2 and 88.2% were achieved when classifying data into 40 labels using the EEGNet and CNN-LSTM networks, respectively. The second approach considered different sample lengths to evaluate the signal intervals using data that were further enhanced in the preprocessing step.

**Figure 6:**
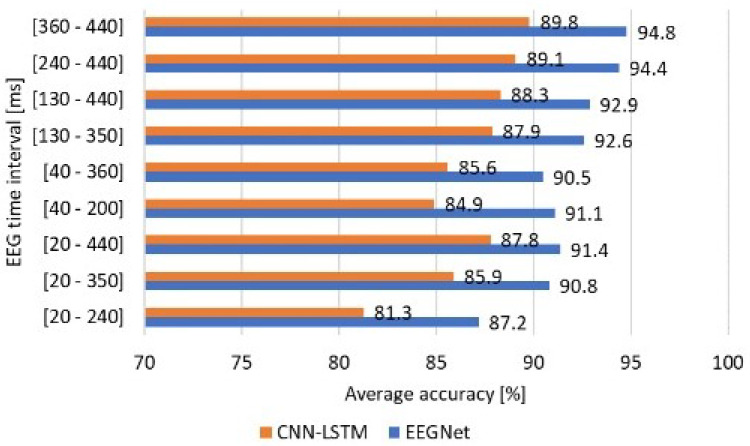
Accuracies achieved when processing data from 54 channels in different EEG time intervals.

Therefore, Figure 6 considers nine data time intervals between 20 and 440 samples. Interval cutting was motivated, firstly, to evaluate the length of 440 samples in small intervals, leaving out data samples at one extremity, and secondly, to compare the outcomes of the suggested method with those of the state of the art (SOTA). Thus, the classification accuracies reported in Figure 6 were averaged from those achieved by each k-fold iteration. The best average accuracy of 94.8% was reached by processing data in the 360–440 ms interval, while the lowest average accuracy of 87.2% was obtained for data in the 20–240 ms interval with the EEGNet model. The CNN–LSTM architecture performed the classification task best when processing the 360–440 ms interval (89.8%) rather than the 20–240 ms interval (81.3%).

In summary, the developed strategy of sample stretching and splitting enabled different outcomes, which is discussed in the next section. Furthermore, the relative diagonal results of the confusion matrices presented in Table 8 and Table 9 illustrate the accuracy of the predicted labels versus true labels for a specific output class. In the aforementioned tables, each column represents the average diagonal results of the 10-fold partial results for the considered time windows. As can be seen, the EEGNet model performed best in data class labeling for the 360–440 ms time window, followed by the 240–440 ms, 130–440 ms, and 130–350 ms intervals, achieving relative accuracies of 91.9, 93.0, 89.8, and 90.9%, respectively. In addition, relative accuracies of 87.9, 86.2, 86.9, and 85.9% were obtained when processing the 360–440 ms, 240–440, 130–440, and 130–350 ms intervals, respectively, with the CNN–LSTM classifier.

In addition to the classification accuracy and confusion matrix metrics, round-robin leave-one-subject-out cross validation (LOSOV) [63] was used to evaluate the approach proposed in this work. The LOSOV method uses data from five subjects to train the classifiers, while those from one subject are utilized for validation in each iteration. Therefore, the data cross-subject variability was evaluated using 9969 samples for model training and 1995 samples for model testing. Table 10 presents the results obtained utilizing the LOSOV method. The EEGNet model achieved 94.1% using data from Subject 3 for testing in the label classification task. In addition, the CNN-LSTM architecture exhibited the best accuracy of 88.2% when testing on Subject 4 data.

### 5.4. Discussion

**Figure 7:**
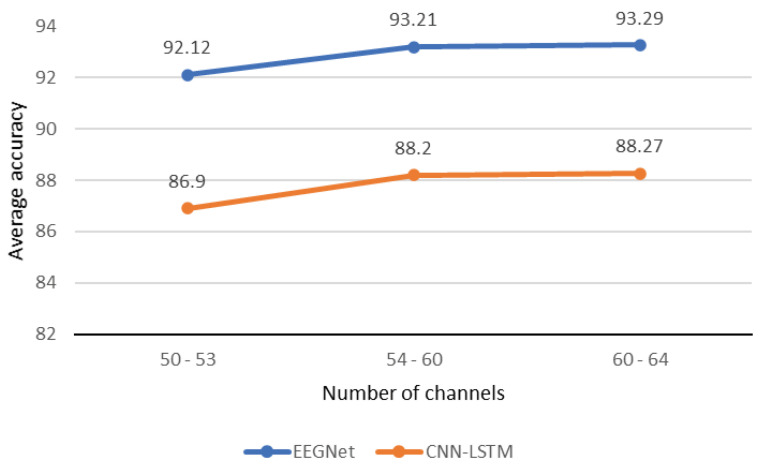
Illustration of the number of channel settings. As shown in the figure, the observable change in the classification accuracy occurred by reducing the number of channels beyond 54 after selecting the original 64.

An important contribution of this work is optimizing the number of contributing channels while ensuring accurate data classification for BCI applications. A subsequent check of the discriminant frames was introduced to deduce the signal lengths that were more enhanced by MNE preprocessing. In this work, we aimed to use less than 50% of the available channels. On the one hand, this addresses a big data processing issue for embedded BCI applications where computational resources are constrained; for instance, see [64]. On the other hand, selecting discriminating channels allowed us to minimize the influence of channels whose intrinsic characteristics did not significantly contribute to the classification process based on the information conveyed [65]. Thus, Algorithm 1 was configured to select 64 channels from the 128 available based on the mutual information obtained using the cross-entropies. After configuring the classifiers, the first tests were used to set Algorithm 1 at a limit of 60 channels, assess the outcomes, and then compare them with those achieved with 64 channels. The difference between outcomes was minimal compared to the large amount of data conserved in the processing chain when considering the 64 channels. Comparing the outcomes of 55 channels with the outcomes of 60 and 64 channels produced the same findings. However, when proceeding with the same approach with 50 and 55 channels, it was discovered that 53 of those selected provided a larger difference than 54 channels as illustrated in Figure 7.

Consequently, for the remaining processing steps, 54 channels were utilized. The accuracy for each number of channels can be illustrated using a figure, such as in [65] where the general trend follows that of a Gaussian curve. In this typical case, the accuracy curve peak corresponds to the optimal number of channels. The remaining labor consisted in optimizing the number of electrodes to 19 or less using the Emotiv Flex equipment to record data from 32 channels. In practice, reducing the number of channels for BCI applications should also take into account the classification accuracy, and, in the case of our method, the parameters required by the classifiers. The construction of contributing sets of channels based on mutual information allowed us to obtain an approximate 48.9% average accuracy with 14 channels, 62.4% with 27 channels, and 77.5% with 39 channels, until the results reported in Figure 7 for 64 channels were obtained. In summary, to the best of our knowledge, no studies in the associated literature produced satisfactory results with fewer than 29 channels with the PL dataset. From the 54 channels selected, 33 were located in the parietal–occipital cortex and 16 in the central cortex. In other words, the mental task of image visualization produced a greater neuronal effect in the motor and visual cortices than in others regions as reported by Zheng and Chen [34]. Thereafter, preprocessing using MNE allowed us to obtain enhanced data that were more suitable for the classifiers. Table 11 presents the obtained results with and without the MNE preprocessing block integration.

In the case of the EEGNet network, an average relative benefit of 12.8% was obtained after implementing the preprocessing stage of the MNE, whereas the CNN-LSTM classifier enabled a relative gain of 13.9%.

The final data classification step was carried out using the EEGNet and CNN-LSTM models. Based on the metrics presented above, a better performance was observed with the EEGNet architecture as compared to the CNN-LSTM model. Essentially built around distinct architectures, the CNN-LSTM model required roughly 107,278 parameters, whilst only 54,632 parameters were used to configure the EEGNet network. That is almost half the number of parameters utilized by the CNN-LSTM model. In addition to the performance, this finding gives credence to the EEGNet network for embedded BCI applications, as a small number of parameters is another essential requirement for the EEGNet model implementation on embedded systems. Furthermore, the k-fold cross-validation method provided better results than those obtained with LOSOV. Without comparing the two validation approaches in this work, and assuming that all subjects performed the experiment well, this discrepancy could be explained by the reduced number of samples used to train the model in the LOSOV approach as compared with the k-fold method. Table 12 compares the outcomes obtained in this work with those reported in the related literature. This is essentially based on the PL dataset, the number of channels, and parameters required by the classifier. With the exception of [34], in which partial tests were performed with 29 channels, the majority of the published comparative results are based on data processed using 128 electrodes. In this work, the number of channels was reduced by approximately 57.8%, i.e., from the 128 used by most benchmarks. In this sense, the last row in Table 12 presents the results obtained using all dataset channels, i.e., by omitting the channel selection block. Therein, comparing the results achieved with 54 channels with those obtained with 128 channels, the difference was approximately 2.6 % and 74 channels, considering the classification accuracy and the number of channels, respectively.

In addition, the two last columns in Table 12 compare the number of parameters with classification accuracies.

Spampinato [22] and Nandini [36] obtained accuracies of 82.9 and 81.59% by implementing the RNN/CNN and STFT+EEGCapsNet models, respectively. The proposed approach with both architectures performed the class labeling task better than the aforementioned models. Xiao [34,35] and Nastaran Khaleghi et al. [37] reported accuracies of 96.27, 97.13, and 98.4% using the Bi-LSTM-AttGW, LSTMs_B, and FC-GDN architectures, respectively. Their achievement exceeded the classification accuracy obtained in this work by an average of 2.4%. However, despite the reduced number of selected channels, i.e., 29, the Bi-LSTM-AttGW model proposed by Zheng and Chen required a larger number of parameters—almost six times the number used to train the EEGNet model. Thus, compared to EEGNet, their model would require more computational resources to implement integrated BCI applications. Since there was a 1.47% accuracy discrepancy (96.27–94.8), it can be inferred that the trade-off for an embedded BCI application consists of using 54 channels with $0.54\times 10^{5}$ parameters or 29 channels with more than $3\times 10^{5}$ parameters. The same findings emerged for the FC-GDN model, which used 11 times more network parameters than EEGNet, in addition to 128 channels. Therefore, in this work, we propose a reliable and high-performance alternative for embedded BCI applications that offers a suitable trade-off between the accuracy and the number of channels and training parameters.

## 6. Conclusions and Future Work

### 6.1. Conclusions

This work aimed to classify visual EEG signals using 40 labels, i.e., addressing the visual EEG multiclass classification challenge, using a smaller number of channels while maintaining a reliable classification accuracy. Public data from the PL dataset, which includes 11,964 EEG signal segments, were used for the experiment. Specifically, a set of 54 discriminating channels was built using a channel selection approach based on mutual information. Thereafter, data were enhanced using the MNE method. In the final stage, the EEGNet and CNN-LSTM architectures served as classifiers to label the data according to the defined classes with an optimized number of training parameters. The results demonstrate that the EEGNet classifier is superior to the CNN-LSTM, achieving the highest accuracy of 94.8%. Compared with the models proposed in the literature, our method incorporates the trade-off between the classification accuracy and the number of channels and parameters, as the latter is a more desirable criterion in the implementation of embedded BCI systems based on EEG signals. However, our study also has certain weaknesses. For instance, the results presented in this work are constrained to the configuration set for the preprocessing and classification blocks. In addition, this study is exclusively based on the data provided by the PL database. The source codes of this project are available to the public at https://github.com/Tatyvelu/EEG-Visual-Multiclass-Classification (accessed on 5 June 2024).

### 6.2. Forthcoming Work

The lack of databases with more than 40 classes, i.e., similar to the PL dataset, motivated us to consider multiclass database construction for future work. This may consist of EEG signals from fruit sequence visualization. Such a dataset would be useful for implementing the EEGNet model in an NAO robot to assist people in fruit-type recognition.

## Figures and Tables

**Table 1:** The use of the PL dataset in the recent literature. Nb.P. refers to the number of network parameters.

Works	Models	Dataset	Channels	Nb.P.	Acc. [%]
Zheng and Chen [34]	Bi-LSTM-AttGW	PL	128	>$3\times 10^{5}$	99.50
Zheng et al. [35]	LSTMS_B	PL	128	–	97.13
Spampinato et al. [22]	RNN/CNN	PL	128	–	82.9
Kumari et al. [36]	STFT + EEGCapsNet	PL	128	–	81.59
Khaleghi et al. [37]	FC-GDN	PL	128	>$6\times 10^{5}$	98.4

**Table 2:** Main layer parameters for the proposed EEGNet model.

Layer (Type)	Output Shape	Parameters
Input Layer	(None, 54, 440, 1)	0
Conv2D	(None, 54, 440, 8)	320
Batch_normalization_1	(None, 54, 440, 8)	32
Depthwise_conv2D	(None, 1, 440, 80)	4320
Batch_normalization_2	(None, 1, 440, 80)	320
Activation_1	(None, 1, 440, 80)	0
Average_pooling2D_1	(None, 1, 110, 80)	0
Dropout_1	(None, 1, 110, 80)	0
Separable_conv2D	(None, 1, 110, 80)	7680
Batch_normalization_3	(None, 1, 110, 80)	320
Activation_2	(None, 1, 110, 80)	0
Average_pooling2D_2	(None, 1, 13, 80)	0
Dropout_2	(None, 1, 13, 80)	0
Flatten	(None, 1040)	0
Dense	(None, 40)	41,640
Softmax	(None, 40)	0
Total number of parameters		54,632

**Table 3:** Parameters summary of the proposed CNN-LSTM model.

Layer (Type)	Output Shape	Parameters
Conv1D_layer1	(None, 440, 128)	20,864
Dropout_1	(None, 440, 128)	0
Activation_1	(None, 440, 128)	0
Max_Pooling	(None, 220, 128)	0
Conv1D_layer2	(None, 220, 64)	24,640
Dropout_2	(None, 220, 64)	0
Activation_2	(None, 220, 64)	0
LSTM_layer1	(None, 220, 64)	33,024
Conv1D_layer3	(None, 220, 64)	12,352
Dropout_3	(None, 220, 64)	0
Activation_3	(None, 220, 64)	0
LSTM_layer2	(None, 32)	12,416
Dropout_4	(None, 32)	0
Dense_1	(None, 54)	1782
Activation_4	(None, 54)	0
Dense_2	(None, 40)	2200
Total number of parameters		107,278

**Table 4:** The number of samples in the visual EEG dataset for each subject.

Order	Subject	Segments Order	Number of Samples
1	4	from 1 to 1995	1995
2	1	from 1996 to 3980	1985
3	6	from 3981 to 5976	1996
4	3	from 5977 to 7972	1996
5	2	from 7973 to 9968	1996
6	5	from 9969 to 11,964	1996
Total	All subjects	from 1 to 11,964	11,964

**Table 5:** The summary of the experiment protocol parameters.

Parameter	Number
Total number of images	2000
Number of images per class	50
Number of classes	40
Display mode	sequential
Display time per image block	0.5 s
Sampling frequency	1000 Hz
Pause time between classes	10 s
Number of sessions	4
Session running time	350 s
Total running time	1400 s

**Table 6:** Channels selected applying Algorithm 1. The term *Nr.Ch.* in the second column refers to the number of channels.

Brain Area	Nr.Ch.	Description
Frontal–Central–Central	3	FCC1h, FCC2h, FCC4h
Frontal–Central	2	FC1, FC2
Central	7	C1, C2, C3, Cz, C4, C5, C6
Central–Parietal	5	CP1, CP2, CP3, CPz, CP4
Central–Central–Parietal	4	CCP1h, CCP2h, CCP3h, CCP4h
Occipital	7	O1, Oz, O2, I1, O11h, O12h, I2
Parietal	8	Pz, P1, P2, P3, P4, P5, P6, P8
Parietal–Occipital	7	PO7, PO3, POz, PO4, PO8, PO9, PO10
Parietal–Parietal–Occipital	6	PPO9h, PPO5h, PPO1h, PPO2h, PPO6h, PPO10h
Parietal–Occipital–Occipital	5	POO1, POO2, POO9h, POO10h, Iz
TOTAL	54	

**Table 7:** Achieved accuracy in each k-fold iteration for the two proposed classifiers with a time interval of 440 samples.

k-Fold	Number of Segments		Classification Accuracy [%]	
	Training	Testing	EEGNet	CNN-LSTM
1	10,768	1197	92.8	88.7
2	10,768	1197	93.1	88.9
3	10,768	1197	92.2	89.1
4	10,768	1197	93.6	87.3
5	10,768	1197	94.3	88.8
6	10,769	1196	93.7	88.2
7	10,769	1196	92.8	87.9
8	10,769	1196	94.1	88.1
9	10,769	1196	92.9	87.5
10	10,769	1196	93.3	88.4
Average			93.2	88.2

**Table 8:** Summary of diagonal results using the confusion matrix to evaluate the performance of the proposed EEGNet model when processing the [130–350], [130–440], [240–440], and [360–440] time windows.

N°	Class	Average Accuracies per Class Label ([%])				Relative
		[130–350]	[130–440]	[240–440]	[360–440]	
1	cats	91.2	89.7	93.0	92.3	91.5
2	sorrels	90.8	90.1	92.8	91.9	91.4
3	elephants	91.0	89.4	93.3	91.8	91.3
4	fish	90.6	90.3	93.2	92.0	91.5
5	dogs	90.8	90.2	92.6	91.9	91.3
6	airliners	91.1	90.3	92.6	92.2	91.5
7	brooms	90.7	89.4	93.1	92.3	91.3
8	pandas	90.9	90.1	92.8	91.8	91.4
9	canoes	91.1	89.9	93.2	91.8	91.5
10	phones	91.3	89.6	92.8	91.9	91.4
11	mugs	90.8	89.7	93.1	91.6	91.3
12	convertibles	90.7	89.6	92.8	91.9	91.2
13	computers	91.0	89.9	92.8	91.7	91.3
14	fungi	91.3	90.2	92.8	92.1	91.6
15	locomotives	91.3	89.8	93.4	91.7	91.5
16	espresso	90.6	89.5	92.8	91.7	91.1
17	chairs	91.2	90.1	93.0	92.2	91.6
18	butterflies	91.0	89.6	93.2	92.4	91.5
19	golf	90.5	89.8	92.7	91.8	91.2
20	piano	91.1	90.0	92.9	92.2	91.5
21	iron	90.7	90.1	93.3	91.6	91.4
22	daisy	90.6	89.4	93.2	91.7	91.2
23	jacks	91.2	89.8	92.9	91.7	91.4
24	mailbags	90.5	89.4	93.4	92.3	91.4
25	capuchin	91.2	90.4	93.3	91.8	91.6
26	missiles	91.1	90.2	93.1	92.4	91.7
27	mittens	90.6	89.6	92.8	91.9	91.2
28	bikes	91.2	90.1	93.2	91.9	91.6
29	tents	90.8	90.1	92.8	92.2	91.4
30	pajama	90.7	90.2	93.3	91.6	91.4
31	parachutes	91.3	90.1	93.0	91.9	91.5
32	pools	90.5	89.7	93.2	91.8	91.3
33	radios	90.9	89.7	92.8	92.3	91.4
34	cameras	91.1	90.3	93.2	91.9	91.6
35	guitar	90.8	90.1	93.0	91.9	91.4
36	guns	91.2	90.1	92.8	92.3	91.6
37	shoes	90.6	89.4	92.7	92.2	91.2
38	bananas	90.8	90.1	93.2	91.9	91.5
39	pizzas	90.8	89.9	92.7	92.3	91.4
40	watches	91.1	89.8	93.2	91.8	91.4
Relative		90.9	89.8	93.0	91.9	91.4

**Table 9:** Overview of diagonal outcomes utilizing the confusion matrix to assess the CNN-LSTM model’s performance when processing the [130–350], [130–440], [240–440], and [360–440] data intervals.

N°	Class	Average Accuracies per Class Label ([%])				Relative
		[130–350]	[130–440]	[240–440]	[360–440]	
1	cats	85.8	86.9	86.3	88.1	86.7
2	sorrels	86.1	87.0	86.2	87.9	86.8
3	elephants	85.8	86.8	86.1	87.9	86.6
4	fish	86.2	87.1	86.5	87.7	86.8
5	dogs	85.9	86.8	86.2	87.9	86.7
6	airliners	86.3	87.1	85.9	88.2	86.8
7	brooms	85.8	87.1	86.3	87.8	86.7
8	pandas	86.1	86.8	86.3	87.6	86.7
9	canoes	85.8	86.7	86.8	87.4	86.6
10	phones	86.2	86.9	86.1	87.7	86.7
11	mugs	85.9	86.8	86.2	88.1	86.7
12	convertibles	86.2	86.9	86.8	87.9	86.9
13	computers	86.1	86.6	86.8	88.1	86.9
14	fungi	85.8	87.1	86.3	87.9	86.7
15	locomotives	85.5	86.8	86.1	87.6	86.5
16	espresso	86.1	86.8	85.7	87.4	86.5
17	chairs	86.3	87.2	86.3	88.2	87.0
18	butterflies	85.7	87.1	86.3	87.4	86.6
19	golf	85.2	86.6	86.7	87.9	86.6
20	piano	85.9	87.2	86.1	89.7	87.2
21	iron	86.1	86.8	85.9	87.4	86.5
22	daisy	86.1	87.2	85.8	87.7	86.7
23	jacks	85.6	86.5	86.1	88.2	86.6
24	mailbags	86.2	87.1	85.8	87.8	86.7
25	capuchin	85.8	86.9	86.3	87.8	86.7
26	missiles	85.9	87.1	86.4	88.3	86.9
27	mittens	86.2	87.1	86.0	88.3	86.9
28	bikes	85.4	86.8	86.4	87.7	86.5
29	tents	85.6	86.8	86.1	88.2	86.6
30	pajama	86.0	87.2	86.1	87.8	86.7
31	parachutes	86.1	86.8	86.3	88.1	86.8
32	pools	85.7	87.1	86.3	87.5	86.6
33	radios	86.2	87.1	85.9	87.7	86.7
34	cameras	85.9	86.9	86.3	88.1	86.8
35	guitar	86.1	87.0	86.2	89.5	87.2
36	guns	85.7	87.1	86.4	87.9	86.7
37	shoes	86.1	87.3	86.2	87.8	86.8
38	bananas	85.7	86.8	86.3	87.8	86.6
39	pizzas	86.0	86.9	86.1	88.2	86.8
40	watches	85.7	86.8	85.9	88.2	86.6
Relative		85.9	86.9	86.2	87.9	86.7

**Table 10:** Classifier performance using the LOSOV method for data in the 360–440 ms time window.

Round	Data from Subject		Classification Accuracy [%]	
	Training	Testing	EEGNet	CNN-LSTM
1	1-2-3-4-5	6	91.7	86.3
2	2-3-4-5-6	1	93.4	87.4
3	3-4-5-6-1	2	92.5	87.9
4	4-5-6-1-2	3	94.1	86.8
5	5-6-1-2-3	4	93.7	88.2
6	6-1-2-3-4	5	92.8	87.3
Average			93.0	87.3

**Table 11:** Results related to preprocessing block ablation.

N°	Interval [ms]	EEGNet Accuracy [%]		CNN-LSTM Accuracy	
		with MNE	without MNE	with MNE	without MNE
1	[130–350]	92.6	80.3	87.9	73.8
2	[130–440]	92.9	79.2	88.3	74.1
3	[240–440]	94.4	81.8	89.1	75.4
4	[360–440]	94.8	82.1	89.8	76.2
Average benefit		12.8		13.9	

**Table 12:** Comparison of the achieved results with those of the state of the art. *Nb.P.* refers to the number of network parameters.

Works	Models	Dataset	Channels	Nb.P.	Acc. [%]
Zheng and Chen [34]	Bi-LSTM-AttGW	PL	29	>$3\times 10^{5}$	96.27
Zheng et al. [35]	LSTMS_B	PL	128	–	97.13
Spampinato et al. [22]	RNN/CNN	PL	128	–	82.9
Kumari et al. [36]	STFT + EEGCapsNet	PL	128	–	81.59
Khaleghi et al. [37]	FC-GDN	PL	128	>$6\times 10^{5}$	98.4
Our method	EEGNet/CNN-LSTM	PL	54	$0.54\times 10^{5}$	94.8
Ablation	EEGNet	PL	128	$0.93\times 10^{5}$	97.4

## Data Availability

Data are available under a formal request.

## Abbreviations

BCI	Brain–Computer Interface
EEG	Electroencephalogram
CSP	Common Spatial Pattern
SVM	Support Vector Machine
DCNN	Deep Convolutional Neural Network
LSTM	Long-Short-Term Memory
fNIRS	Functional near-infrared spectroscopy
SD	Source Detector
RNN	Recurrent Neural Network
CNN	Convolutional Neural Network
EEGNet	Compact convolutional neural network for EEG-based BCI
PL	Perceive Lab
CNN-LSTM	Convolutional Neural Network–Long Short-Term Memory
ERP	Event-Related Potential
STFT	Short-Term Fourier Transform
VEP	Visual-evoked potentials
SSVEP	Steady-State Visually Evoked Potentials
KNN	K-Nearest Neighbors
EEGCapsNet	Capsule Network
FC-GDN	Functional Connectivity-Based Geometric Deep Network
MutIn	Mutual Information
MNE	Minimum-Norm Estimates software suite
MI	Motor Imagery
MI-EEG	Motor Imagery EEG
EVC	Visual EEG Classification
EMG	Electromyogram
KLD	Kullback–Leibler Divergence
CLR	Cyclical Learning Rate
SOTA	State of the Art
LOSOV	Round-Robin Leave-One-Subject-Out Cross-Validation
